# PTPN21/Pez Is a Novel and Evolutionarily Conserved Key Regulator of Inflammation *In Vivo*

**DOI:** 10.1016/j.cub.2020.11.014

**Published:** 2021-02-22

**Authors:** Jennie S. Campbell, Andrew J. Davidson, Henry Todd, Frederico S.L.M. Rodrigues, Abigail M. Elliot, Jason J. Early, David A. Lyons, Yi Feng, Will Wood

**Affiliations:** 1 Centre for Inflammation Research, University of Edinburgh, Queens Medical Research Institute, 47 Little France Crescent, Edinburgh BioQuarter, Edinburgh EH16 4TJ, UK; 2 School of Cellular and Molecular Medicine, Faculty of Biomedical Sciences, University of Bristol, Bristol BS8 1TD, UK; 3 Centre for Discovery Brain Sciences, University of Edinburgh, Edinburgh, UK

**Keywords:** *Drosophila*, zebrafish, inflammation, migration, neutrophil, macrophage, Draper, Pez, PTPN21, Megf10

## Abstract

*Drosophila* provides a powerful model in which to study inflammation *in vivo*, and previous studies have revealed many of the key signaling events critical for recruitment of immune cells to tissue damage. In the fly, wounding stimulates the rapid production of hydrogen peroxide (H_2_O_2_).^1^^,^^2^ This then acts as an activation signal by triggering a signaling pathway within responding macrophages by directly activating the Src family kinase (SFK) Src42A,^3^ which in turn phosphorylates the damage receptor Draper. Activated Draper then guides macrophages to the wound through the detection of an as-yet unidentified chemoattractant.^3^, ^4^, ^5^ Similar H_2_O_2_-activated signaling pathways are also critical for leukocyte recruitment following wounding in larval zebrafish,^6^, ^7^, ^8^, ^9^ where H_2_O_2_ activates the SFK Lyn to drive neutrophil chemotaxis. In this study, we combine proteomics, live imaging, and genetics in the fly to identify a novel regulator of inflammation *in vivo*; the PTP-type phosphatase Pez. Pez is expressed in macrophages and is critical for their efficient migration to wounds. Pez functions within activated macrophages downstream of damage-induced H_2_O_2_ and operates, via its band 4.1 ezrin, radixin, and moesin (FERM) domain, together with Src42A and Draper to ensure effective inflammatory cell recruitment to wounds. We show that this key role is conserved in vertebrates, because “crispant” zebrafish larvae of the Draper ortholog (MEGF10) or the Pez ortholog (PTPN21) exhibit a failure in leukocyte recruitment to wounds. This study demonstrates evolutionary conservation of inflammatory signaling and identifies MEGF10 and PTPN21 as potential therapeutic targets for the treatment of inflammatory disorders.

## Highlights

• Pez is a novel regulator of inflammation needed for macrophage recruitment to wounds
• During *Drosophila* inflammation, Pez acts in the H_2_O_2_/Src42a/Draper signaling axis
• Pez and Draper form dynamic clusters within macrophages in response to tissue damage
• Pez (PTPN21) and Draper (MEGF10) orthologs play conserved roles in fish leukocytes

## Teaser

Through the combination of proteomics, genetics, and live imaging, Campbell et al. identify Pez as a novel regulator of inflammation *in vivo*. Pez acts in the damage-sensing, H_2_O_2_/Src42a/Draper signaling axis, wherein it dynamically clusters with Draper to enable rapid leukocyte migration to epithelial wounds in both the fly and fish.

## Results and Discussion

To identify further components of the H_2_O_2_-Src42A-Draper inflammatory signaling axis in *Drosophila* macrophages, we undertook a phosphoproteomics approach to identify phosphoproteins regulated downstream of H_2_O_2_ and Src42A. Control and *src42A*^*[E1]*^ mutant stage 15 embryos were disaggregated by crushing to engage global inflammatory signaling (Figure S1A). Disaggregation was carried out both with or without catalase (to quench H_2_O_2_ signaling), and GFP-positive macrophages (*srp-Gal4* driven upstream activating sequence [UAS]-GFP) were collected by fluorescence-activated cell sorting (FACS). The macrophage-specific peptides obtained were tandem mass tagged (TMT) labeled, phospho-enriched, and identified by liquid chromatography-mass spectrometry (Figure S1B). Finally, an organism-specific database search was conducted to identify the peptides isolated (Figures S1C–S1E). This revealed the protein tyrosine phosphatase (PTP)-type phosphatase Pez as differentially phosphorylated in the presence of both H_2_O_2_ and Src42A (Figures S1E and S1F). Because the ortholog of Pez (PTPN21) had previously been identified as an interactor and regulator of SFK signaling in other contexts,^10^, ^11^, ^12^ we chose to investigate Pez in inflammatory cell migration.

**Figure 1:**
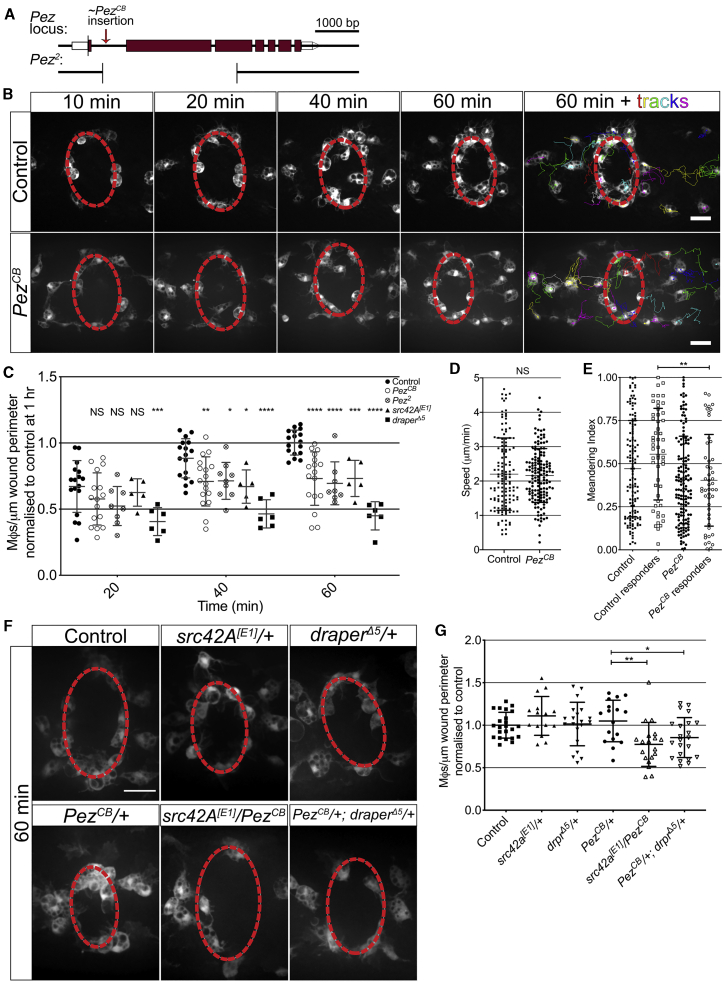
Pez Is Required for Macrophage Migration to Epithelial Wounds and Functions within the H_2_O_2_-Src42A-Draper Signaling Pathway (A) Pez locus highlighting mutant alleles. Approximate CB insertion (6.056 kb) site is indicated. *Pez*^*2*^ deletion is marked below, adapted from Poernbacher et al.^17^ (B) Live imaging of inflammation following laser ablation reveals reduced macrophage recruitment in *Pez*^*CB*^ mutants. Wound margin is denoted by dashed red line. Cell tracks are shown at 1 h. (C) Quantification reveals a significant decrease in macrophage numbers at wounds in the two *Pez* mutant lines at 40 and 60 min post-injury (n ≥ 10 wounded embryos/genotype; multiple t tests with Holm-Sidak multiple comparisons). (D and E) Cell tracking reveals (D) macrophage speed post-wounding is unaffected in *Pez*^*CB*^ mutants (n ≥ 130 cells from ≥5 embryos/genotype; Mann-Whitney U test), and (E) meandering index is significantly reduced in responding (cells that reach the wound site at any point within 2 h) *Pez*^*CB*^ macrophages (n = 53 responders from ≥5 embryos/genotype; Mann-Whitney U test). (F) Heterozygote (*src42A*^*[E1]*^*/+*, *draper*^*Δ5*^*/+*, and *Pez*^*CB*^*/+*) and transheterozygote (*src42A*^*[E1]*^*/Pez*^*CB*^ and *Pez*^*CB*^*/+; draper*^*Δ5*^*/+*) mutant embryos at 60 min post-wounding. Wound margin is denoted by dashed red line. (G) Significantly reduced macrophage wound recruitment in transheterozygotes embryos versus *Pez*^*CB*^*/+* (n ≥ 15 wounded embryos/genotype; one-way ANOVA with multiple comparisons). All error bars are mean ± SD. NS, not significant; ^∗^p < 0.05, ^∗∗^p < 0.01, ^∗∗∗^p < 0.005, and ^∗∗∗∗^p < 0.001. All scale bars represent 20 μm. See also Figure S2 and Videos S1, S2, and S3.

To determine whether Pez is expressed in embryonic macrophages, we used *Pez-Gal4 (P{GawB}Pez*^*NP4748*^*)* to drive *UAS-GFP* and investigated GFP expression by immunofluorescence. Co-labeling with anti-singed (a macrophage marker in *Drosophila*)^13^ confirmed that Pez is expressed within macrophages at stage 15 of development (Figure S1G). We next sought to determine whether Pez plays a role in normal macrophage behavior using two independent *Pez* mutant lines (Figure 1A). Following their specification from the head mesoderm, macrophages follow a stereotypical migration pattern to become evenly distributed by the end of embryogenesis.^14^, ^15^, ^16^ This characteristic developmental dispersal of macrophages in *Pez* mutant embryos occurred normally, with macrophages following the expected dispersal routes at identical migratory speeds to controls (Figures S1H and S1I; Video S1).

Video S1. Developmental Migration Is Unaffected by Loss of Pez, Related to Figures 1 and S1 Time-lapse imaging of control (LEFT), *Pez^2^* (MIDDLE) and *Pez*^*CB*^ (RIGHT) macrophages during their developmental dispersal (embryonic stage 12 to late stage 14). All macrophages labeled with cytosolic GFP. Macrophages migrate down the entire length of the ventral midline before rapidly dispersing laterally to spread evenly across the embryo. This stereotypical migration pattern is unperturbed in *Pez* mutants. Images acquired at 30 s intervals. The time is shown in minutes and the scale bar is 20 μm.

During this migration, macrophages actively clear developmentally generated apoptotic corpses, which are identifiable inside GFP-expressing macrophages as fluorescent-negative vacuoles.^18^ Live imaging of *Pez* mutant macrophages at stage 15 revealed normal cell morphology, with cells displaying lamellipodial protrusions and containing intracellular vacuoles (Figure S1J; Video S2). Quantification of vacuole numbers in *Pez* mutant macrophages revealed no significant defect in their phagocytic capability (Figure S1K). Finally, live imaging revealed that, following the completion of their dispersal, *Pez* mutant macrophages migrate at the same speed and in the same manner as control cells (Figures S2A–S2C; Video S2). Together, this demonstrates that Pez is dispensable for basal macrophage migration and function.

Video S2. Migration and Morphology of Stage 15 Macrophages Is Normal in *Pez* Mutants, Related to Figures 1, S1, and S2 Time-lapse imaging of basal (unwounded) control (TOP) and *Pez*^*CB*^ (BOTTOM) macrophage migration at embryonic stage 15 (post-dispersal). All macrophages labeled with cytosolic GFP and the Fire LUT is used to overcome saturating GFP signal in the cell body. Macrophages in both control and *Pez*^*CB*^ embryos display dynamic lamellipodial protrusions and contain abundant vacuoles within their cell bodies (fluorescent negative areas within the body of the cell). Images acquired at 30 s intervals. The time is shown in minutes and the scale bar is 20 μm.

To investigate whether Pez plays a role in the inflammatory recruitment of macrophages to wounds, we carried out live imaging following laser ablation. In control animals, this leads to a rapid recruitment of macrophages to the wound site, with numbers peaking 1 h after insult (Figures 1B and 1C). Macrophage counts 1 h post-injury (1 hpi) revealed a significant reduction in macrophage recruitment in both *Pez*^*CB*^ and *Pez^2^* mutant embryos when compared to controls (Figures 1B and 1C). This was despite there being significantly more macrophages within *Pez* mutant embryos (Figure S2D). Importantly, *Pez* mutant wounds closed at comparable rates to controls (Figure S2E). Interestingly, the *Pez* wound recruitment phenotype is comparable to that observed following loss of Src42a (Figure 1C).

To further investigate this inflammatory defect, *Pez*^*CB*^ mutant macrophages were tracked following live imaging (Video S3). This revealed that the reduction in the number of macrophages present at wounds in *Pez*^*CB*^ mutants was not due to a slower inflammatory migration speed (Figure 1D) but due to a lower meandering index in responding cells (Figure 1E). This corresponded to a later arrival time and lower wound residency of macrophages in *Pez*^*CB*^ mutants when compared to controls (Figures S2F and S2G).

Video S3. The Inflammatory Recruitment of Macrophages to Epithelial Wounds Is Perturbed in *Pez*^*CB*^ Mutants, Related to Figure 1 Time-lapse imaging of control (TOP) and *Pez*^*CB*^ (BOTTOM) stage 15 macrophages in the immediate aftermath of wounding by laser ablation. The wound center is denoted by an asterisk (^∗^) in the initial frame, and the wound perimeter is outlined in the final frame. All macrophages labeled with cytosolic GFP and the Fire LUT is used to overcome saturating GFP signal in the cell body. Videos show impaired recruitment in *Pez*^*CB*^ mutant such that by 60 minutes there is a visible difference in recruited macrophages in comparison to the control. Images acquired at 30 s intervals. The time is shown in minutes and the scale bar is 20 μm.

Because we initially sought to identify novel interactors in the H_2_O_2_/Src42A/Draper inflammatory signaling pathway and the *Pez* phenotype is comparable to that of Src42a mutants and consistent with a macrophage navigational defect, genetic interaction studies were employed to determine whether Pez lies within the same signaling axis. We found no defect in macrophage recruitment to wounds made in heterozygous *src42A*^*[E1]*^*/+*, *draper*^*Δ5*^*/+*, or *Pez*^*CB*^*/+* embryos. However, wounds made to *src42A*^*[E1]*^*/Pez*^*CB*^ or *Pez*^*CB*^*/+; draper*^*Δ5*^*/+* embryos showed a significant reduction in the number of macrophages recruited at 1 hpi when compared to *Pez*^*CB*^*/+* heterozygotes (Figures 1F and 1G). Taken together, these data demonstrate that Pez is a novel component of the H_2_O_2_/Src42A/Draper signaling pathway and drives macrophage recruitment to wounds.

**Figure 2:**
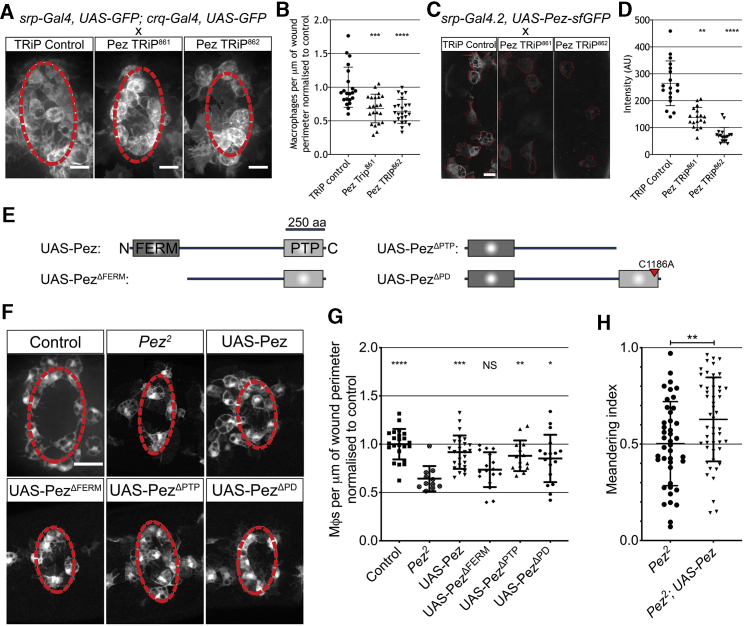
The Role of Pez in Macrophage Wound Recruitment Is Cell Autonomous and Dependent upon the FERM Domain (A) Macrophage-specific expression of Pez-RNAi (TRiP constructs) impairs inflammatory recruitment to wounds (images 1 h post-wounding). Scale bars represent 10 μm. Wound margin is denoted by dashed red line. (B) Pez-RNAi significantly reduces macrophage recruitment to wounds compared to control (n ≥ 21 wounded embryos/genotype; Kruskal-Wallis with Dunn’s multiple comparisons). (C) Pez-sfGFP expression in macrophages (outlined in red) co-expressing either control RNAi or either Pez-RNAi. Scale bars represent 10 μm. (D) Both RNAi lines significantly reduce macrophage Pez-sfGFP intensity levels (n = 18 cells from 6 embryos/genotype; Kruskal-Wallis with Dunn’s multiple comparisons). (E) UAS-Pez expression constructs. FERM domain and PTP domains noted and deletions depicted. For phosphatase dead construct (*UAS-Pez*^*ΔPD*^), the mutated cysteine is noted. Adapted from Poernbacher et al.^17^ (F) Images of wounded *Pez^2^* embryos with macrophage-specific expression of indicated Pez constructs, 1 h post-ablation. Scale bar represents 20 μm. Wound margin is marked by dashed red line. (G) Macrophage-specific expression of *UAS-Pez*, *UAS-Pez*^*ΔPD*^, and *UAS-Pez*^*ΔPTP*^ (but not Pez^ΔFERM^) is sufficient to rescue *Pez^2^* wound recruitment defect (n ≥ 13 wounded embryos/genotype; one-way ANOVA with Dunnett’s multiple comparisons to *Pez^2^*). (H) Quantification of meandering index reveals specific expression of Pez rescues the inflammatory chemotaxis of *Pez*^*2*^ macrophages (n ≥ 42 cells from n ≥ 5 wounded embryos/genotype; unpaired t test). All error bars are mean ± SD. ^∗^p < 0.05, ^∗∗^p < 0.01, ^∗∗∗^p < 0.005, and ^∗∗∗∗^p < 0.001. See also Figure S2 andVideo S4.

Because Pez is widely expressed in stage 15 embryos (Figure S1G), we next confirmed that the role of Pez in macrophage wound recruitment was cell autonomous. To achieve this, we used two macrophage-specific drivers (*srp-gal4* and *crq-gal4*) to express one of two Pez-specific RNAi constructs and quantified macrophage recruitment to wounds. Macrophage-specific Pez RNAi led to a significant reduction in the number of macrophages at epithelial wounds at 1 hpi, demonstrating that Pez is required within macrophages for effective chemotaxis (Figures 2A and 2B). These RNAi constructs were validated and were sufficient to significantly reduce Pez protein levels *in vivo* (Figures 2C and 2D).

We next sought to investigate the mechanism by which Pez is acting within chemotaxing macrophages. As well as a PTP domain, Pez harbors an N-terminal FERM domain (Figure 2E). To determine which domain of Pez is functional during macrophage recruitment, we expressed truncated Pez constructs^17^ in macrophages alongside GFP in a *Pez*^*2*^ mutant background (Figures 2E and 2F). We re-expressed four Pez constructs in *Pez*^*2*^ mutant macrophages—full-length Pez (*UAS-Pez*), Pez lacking the FERM domain (*UAS-Pez*^*ΔFERM*^), Pez lacking the PTP domain (*UAS-Pez*^*ΔPTP*^), and a phosphatase-dead Pez construct (*UAS-Pez*^*ΔPD*^). As expected, macrophage-specific expression of the full-length construct rescued both the wound recruitment and chemotaxis defect seen at 1 hpi in *Pez*^*2*^ mutants (Figures 2F–2H). Interestingly, expression of either of the phosphatase mutant constructs also rescued the mutant phenotype (Figures 2F and 2G). However, the ability of *Pez* mutant macrophages to migrate to wounds was not restored following the expression of *UAS-Pez*^*ΔFERM*^—demonstrating a specific requirement for the FERM domain of Pez in driving macrophage wound recruitment (Figures 2F and 2G; Video S4). Intriguingly, it is the FERM domain of the human Pez ortholog PTPN21 that has been demonstrated to directly bind to Src family kinases.^12^

Video S4. The Wound Recruitment Defect in *Pez*2 Mutants Can Be Rescued by the Expression of Pez, but Not Pez^ΔFERM^, Related to Figure 2 Time-lapse imaging of stage 15 macrophages in the immediate aftermath of wounding by laser ablation. The wound center is denoted by an asterisk (^∗^) in the initial frame, and the wound perimeter is outlined in the final frame. Videos show defective recruitment in *Pez*2 mutant (TOP) which is rescued by the macrophage-specific expression of full-length Pez (MIDDLE). However, re-expression of Pez lacking its FERM domain is unable to restore inflammatory recruitment of macrophages (BOTTOM). In addition to the indicated constructs, all macrophages also expressed cytosolic GFP. Images were acquired at 30 s intervals. The time is shown in minutes and the scale bar is 20 μm.

**Figure 3:**
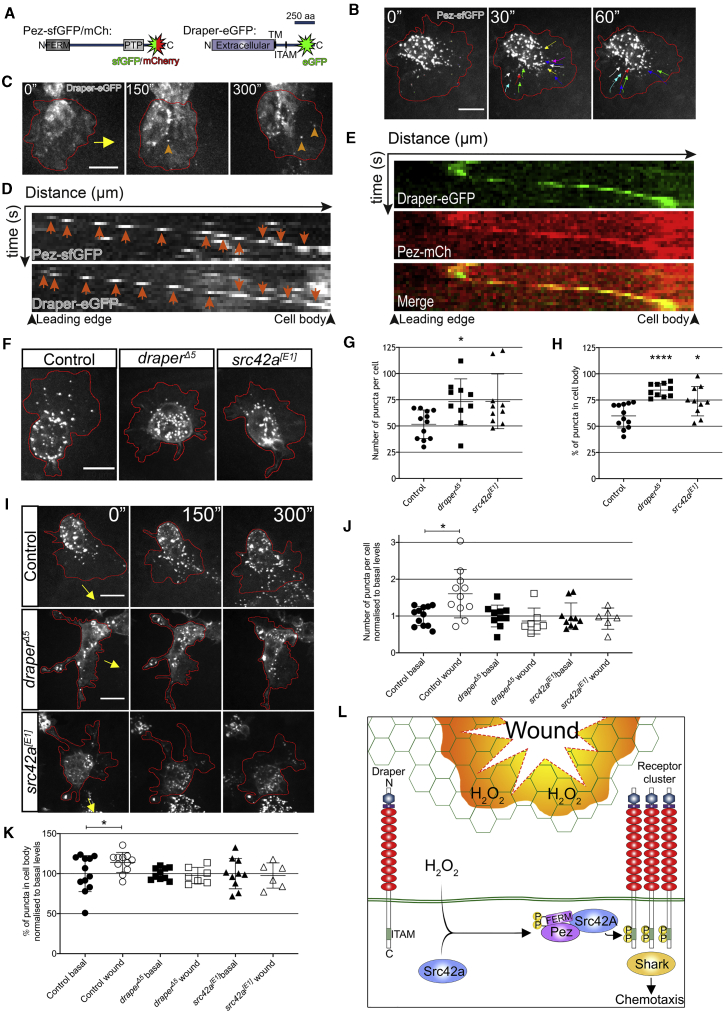
Dynamic Pez Puncta Are Stimulated upon Wounding in a Draper-Dependent Manner (A) Diagrams of fluorescently tagged Pez and Draper constructs. For Pez, the FERM and PTP domains are shown. For Draper, the N-terminal extracellular domain is noted, along with the transmembrane domain (TM) and immunoreceptor tyrosine activation motif (ITAM). (B) Pez forms puncta within the cell body and lamellipod. Dynamic lamellipodial puncta flow inward from the cell periphery (denoted by red line). Colored arrows show puncta tracking over 1 min. (C) Dynamic Draper-EGFP puncta (orange arrowheads) induced post-wounding. Red line donates cell periphery; yellow arrow indicates direction of wound. (D) Kymographs of individual Pez-sfGFP and Draper-EGFP puncta (orange arrows) following wounding demonstrate similar dynamics over time. (E) Kymograph of Draper-EGFP punctum reveals colocalization with Pez-mCh following wounding. For all kymographs, the x axes represent distance starting at lamellipod leading edge (174 nm/pixel; 17.4 μm total). The y axes represent time (10 s/pixel; 2.5 min total). (F) Lamellipodial Pez-sfGFP puncta are suppressed in *draper*^*Δ5*^ and *src42A*^*[E1]*^ mutant macrophages. (G and H) Puncta number (G) and distribution (cell body versus lamellipod; H) significantly altered in mutants (n ≥ 10 cells from ≥5 embryos/genotype; Kruskal-Wallis with Dunn’s multiple comparisons and one-way ANOVA with Tukey’s comparisons, respectively). (I) Images of control, *draper*^*Δ5*^, and *src42A*^*[E1]*^ mutant macrophages (red outlines) 5 min post-wounding. Direction of wound marked by yellow arrow. (J and K) Analysis of Pez puncta 5 min post-wounding reveals (J) a wound-induced significant increase in puncta number that is dependent on both Draper and Src42A (n ≥ 6 cells from ≥5 embryos/genotype for each condition; Kruskal-Wallis with Dunn’s multiple comparisons) and (K) a wound-induced significant increase in the proportion of puncta residing within the cell body of control cells that is absent in *draper* and *src42A* mutants (n ≥ 6 cells from ≥5 embryos/genotype for each condition; one-way ANOVA with Sidak’s multiple comparisons). All error bars are mean ± SD. ^∗^p < 0.05, ^∗∗^p < 0.01, and ^∗∗∗∗^p < 0.001. All scale bars represent 10 μm. (L) Proposed role of Pez in wound-induced Draper clustering. Under basal conditions, Draper’s ITAM domain remains in an inactive state. Following H_2_O_2_-mediated Src42A activation, phosphorylated Pez is recruited to Draper clusters via its FERM-domain-mediated interaction with Src42a. Acting as an adaptor, Pez coordinates inflammatory Draper signaling via effectors such as Shark, leading to efficient macrophage chemotaxis. See also Figure S2 and Videos S5 and S6.

As FERM domains are involved in protein localization,^19^ we generated tagged *UAS-Pez* constructs to investigate Pez dynamics in macrophages *in vivo* (Figure 3A). Macrophage-specific expression of Pez-sfGFP was sufficient to rescue recruitment to wounds in a *Pez^2^* mutant (Figure S2H), and live imaging of Pez-sfGFP-expressing macrophages revealed dynamic puncta that formed within the lamellipod of macrophages before rapidly shuttling back toward the cell body at a rate of 0.12 ± 0.01 μm/s (Figure 3B). Upon wounding, this process was dramatically stimulated in the lamellipods of macrophages undergoing inflammatory chemotaxis (Video S5), resulting in a transient pulse of lamellipodial Pez puncta in macrophages within the vicinity of the wound, which then collectively flowed into the cell body.

Video S5. Pez Puncta Formation Is Stimulated following Wounding, Related to Figure 3 Time-lapse imaging of Pez-sfGFP expressing, stage 15 macrophages in the immediate aftermath of wounding by laser ablation. The wound center is denoted by an asterisk (^∗^) in the first frame. Wounding stimulates Pez puncta formation (arrows) in lamellipods of responding macrophages. Images were acquired at 10 s intervals. The time is shown in minutes and the scale bar is 20 μm.

Draper has also been shown to cluster into mobile puncta in *Drosophila* macrophage cell lines—a process that is proposed to drive its activation cycle akin to the mammalian T cell receptor.^20^ In order to investigate whether this occurs *in vivo*, we expressed Draper-EGFP in macrophages and visualized its localization through live imaging (Figure 3C). Limited Draper puncta were observed under basal conditions within the cell body of migrating macrophages. However, upon wounding, Draper puncta were observed forming at the leading edge of the lamellipod and flowing back toward the cell body (Figure 3D; Video S6)—which was highly reminiscent of that observed with Pez-sfGFP (Figures 3B and 3D). Co-expression of Draper-EGFP and Pez-mCherry revealed a clear colocalization of these two proteins at wound-induced puncta (Figures 3E and S2I).

Video S6. Draper Puncta Are Stimulated following Wounding, Related to Figures 3 and S2 Time-lapse imaging of stage 15 macrophages expressing Draper-eGFP in the immediate aftermath of wounding by laser ablation. The wound site is denoted by an asterisk (^∗^) in the first frame. Approximately 4 minutes post wounding, Draper puncta formation (arrow) is near synchronously stimulated at leading edge, followed by their inward flow toward the cell body. Images acquired at 10 s intervals. The time is shown in minutes and the scale bar is 20 μm.

We next investigated the localization of fluorescently tagged Draper or Pez in *Pez*, d*raper*, and s*rc42A* mutants. Pez was not necessary for Draper puncta, consistent with the multimerization of Draper driving receptor clustering and implying that Pez instead plays a role in downstream signaling (Figure S2J). In the absence of either Draper or Src42A, macrophages under basal (unwounded) conditions retained Pez-sfGFP puncta, albeit with a slight increase in the absolute number of puncta per cell in *draper* mutant macrophages (Figures 3F and 3G). However, when compared to controls, the dynamic subcellular localization of Pez was strongly perturbed in both these mutants, wherein the Pez puncta were predominantly sequestered in the cell body (Figures 3F and 3H). Furthermore, in response to wounding, there was no stimulation of Pez clustering in either *draper* or *src42A* mutant macrophages as observed in control cells (Figures 3I–3K). These data imply that Pez dynamically relocalizes to the lamellipod in response to wound-induced Draper clustering and Src42a activity in order to potentiate inflammatory signaling.

Importantly, the few remaining lamellipodial Pez puncta within *draper* and *src42A* mutant macrophages appeared to behave normally and flowed toward the cell body with similar dynamics to those in controls (Figure S2K). This, together with the high basal number of Pez puncta present in either mutant relative to control, and the basal clustering of Pez in the control in the absence of detectable Draper puncta, is consistent with Pez having targets other than Draper. However, in response to the wound-induced surge in Draper clustering, Pez is co-opted into these puncta via its FERM-domain-mediated interaction with Src42a. The absence of any role for Pez’s catalytic activity in the Draper-mediated inflammation suggests that Pez is acting as an adaptor protein at Draper clusters. As such, Pez organizes these clusters into effective signaling hubs, allowing the critical threshold of activity to be met in order to drive inflammatory recruitment (Figure 3L).

Having identified a novel regulator of damage-induced inflammation in *Drosophila*, we sought to determine whether the activity of Pez in regulating chemotaxis is conserved in the vertebrate. We therefore investigated both the ortholog of Pez—PTPN21—and the ortholog of Draper—MEGF10—in a zebrafish leukocyte wound recruitment model. First, to confirm whether PTPN21 and MEGF10 are expressed in larval zebrafish leukocytes, we mined existing RNA sequencing (RNA-seq) datasets for transcript expression.^21^^,^^22^ This revealed that both *ptpn21* and *megf10* transcripts were enriched within neutrophils by 3 days post-fertilization (dpf) and macrophages by 2 dpf (Figures S2L and S2M).

**Figure 4:**
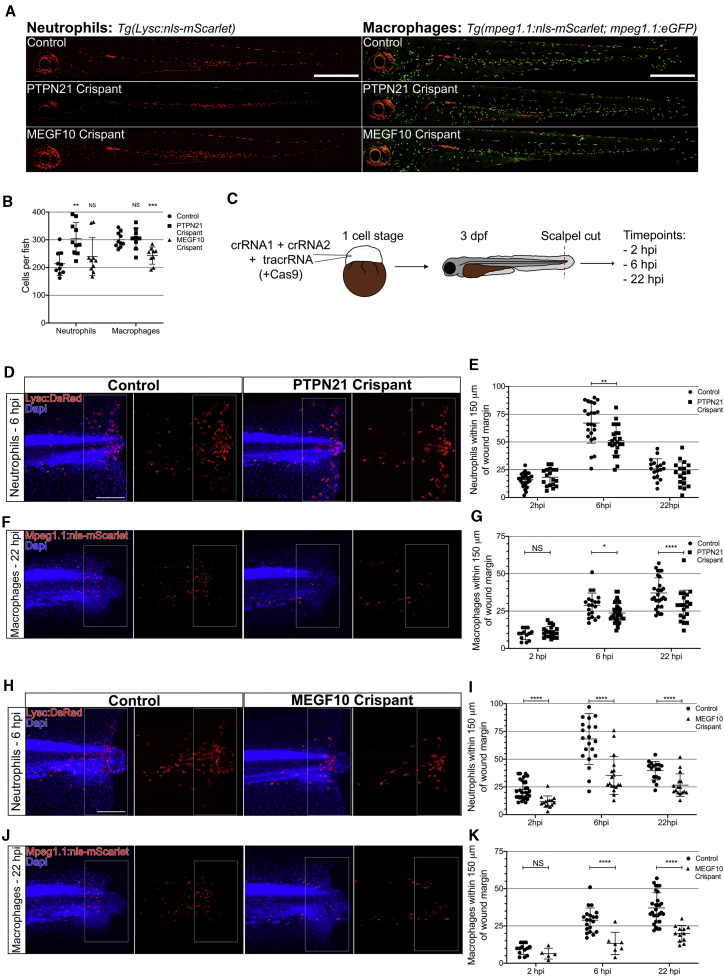
The Orthologs of Pez (PTPN21) and Draper (MEGF10) Are Required for Leukocyte Recruitment to Wounds in Zebrafish Larvae (A) Representative images of entire control, PTPN21 crispant, and MEGF10 crispant zebrafish 3 dpf larvae expressing either *lysc:nls-mScarlet* (neutrophil marker) or *mpeg1.1:nls-mScarlet* and *mpeg1.1eGFP* (macrophage marker). Scale bars represent 500 μm. (B) Quantification of leukocyte numbers revealed an increase in neutrophils in PTPN21 crispants (n = 10 larvae/genotype; Kruskal-Wallis with Dunn’s multiple comparisons) and a decrease in macrophage in MEGF10 crispants (n ≥ 7 larvae/genotype; one-way ANOVA with Dunnett’s multiple comparisons). (C) For wound studies, zebrafish embryos (one cell stage) were injected with 2 CRISPR guide RNAs (crRNAs) alongside tracrRNA and raised to 3 dpf. Following tailfin transection, fish were stained at 2, 6, and 22 h post-injury (hpi). (D) Images of wounded control larvae and PTPN21 crispants at 2, 6, and 22 hpi time points. *Tg(lysC:DsRed)* (red) zebrafish co-stained with DAPI (blue) are shown. Quantification zone of 150 μm proximal to the wound margin is marked by the white box across all images. (E) Significantly reduced neutrophils recruited to the wound at 6 hpi in PTPN21 crispant larvae compared to control (n ≥ 18 wounded larvae/genotype for each time point; multiple t test). (F) Images of wounded control larvae and PTPN21 crispants at 2, 6, and 22 hpi time points. *Tg(mpeg1.1:nls-mScarlet)* (red) zebrafish co-stained with DAPI (blue) are shown. Quantification zone of 150 μm proximal to the wound margin is marked by the white box across all images. (G) Significantly reduced macrophages recruited to the wound at 6 hpi and 22 hpi in PTPN21 crispants (n ≥ 17 wounded larvae/genotype for each time point; multiple t test). (H) Images of wounded control larvae and MEGF10 crispants at 2, 6, and 22 hpi. *Tg(lysc:DsRed)* (red) zebrafish stained with DAPI (blue) are shown. Quantification zone of 150 μm proximal to the wound margin is marked by the white box across all images. (I) Significantly reduced neutrophils recruited to the wound at all time points in MEGF10 crispant compared to the control (n ≥ 15 wounded larvae/genotype for each time point; multiple t test). (J) Images of wounded control larvae and MEGF10 crispants at 2, 6, and 22 hpi time points. *Tg(mpeg1.1:nls-mScarlet)* (red) zebrafish co-stained with DAPI (blue) are shown. Quantification zone of 150 μm proximal to the wound margin is marked by the white box across all images. (K) Significantly reduced macrophages recruited to the wound at 6 hpi and 22 hpi in PTPN21 crispant (n ≥ 13 wounded larvae/genotype for each time point; multiple t test). All error bars are mean ± SD. ^∗^p < 0.05, ^∗∗^p < 0.01, and ^∗∗∗∗^p < 0.001. All scale bars represent 100 μm. See also Figure S2.

To investigate what effects the loss of PTPN21 and MEGF10 have on the development of zebrafish leukocytes, we independently utilized the transgenic neutrophil line *Tg(lysC:NLS-mScarlet)*^23^ and macrophage reporter lines *Tg(mpeg1.1:NLS-mScarlet)* and *Tg*(*mpeg1.1:EGFP*) to generate CRISPR-Cas9-mediated mutant larvae (“crispants”; Figures S2N and S2P). Using restriction fragment length polymorphism analysis (RFLP),^24^ we were able to validate the successful generation of F0 crispant larvae (Figures S2O and S2Q). Imaging the entirety of the crispant fish revealed leukocyte distribution was unaltered when compared with wild type (Figure 4A). However, we found an increase in neutrophil number in PTPN21 crispants—akin to the macrophage phenotype we identify in *Drosophila*—and a 20% reduction in macrophage numbers in MEGF10 crispant fish (Figure 4B).

We next investigated leukocyte recruitment to tailfin transection wounds made in 3 dpf control and crispant embryos (Figure 4C). In control animals, these large wounds trigger a robust inflammatory response—with neutrophil recruitment peaking at 6 hpi and remaining at the wound until 24 hpi and macrophage numbers continuing to increase over a 24 h period.^25^ Consistent with our findings in the fly, wounds made to PTPN21 crispant fish revealed a significant reduction in the peak number of neutrophils recruited to tail fin wounds at 6 hpi (Figures 4D and 4E) and a reduction in macrophage numbers at both 6 hpi and 22 hpi (Figures 4F and 4G).

Because Pez and Draper work together to drive inflammation in *Drosophila* macrophages, we investigated whether MEGF10 is also required for leukocyte recruitment to wounds. Indeed, neutrophils in MEGF10 crispants showed a significantly reduced wound recruitment as early as 2 h post-wounding, and in macrophages, MEGF10 crispant showed nearly 50% reduction at 6 and 22 h post-wounding (Figures 4H–4K). This provides compelling evidence that both PTPN21 and MEGF10 regulate inflammation in zebrafish and that the H_2_O_2_-Src42A-Pez-Draper signaling axis is an evolutionarily conserved signaling pathway that directs the earliest innate immune inflammatory response to damage *in vivo*. Further studies are required to identify more components of this inflammatory signaling axis, but from this study, PTPN21 and MEGF10 emerge as key regulators of inflammation and should now be explored as potential therapeutic targets for the treatment of inflammatory disorders.

## STAR★Methods

### Key Resources Table

**Table:** 

REAGENT or RESOURCE	SOURCE	IDENTIFIER
**Antibodies**		

α-GFP	Abcam	Abcam Cat# ab13970; RRID: AB_300798
α-singed	DSHB	DSHB Cat# sn 7C; RRID: AB_528239
α-armadillo	DSHB	DSHB Cat# N2 7A1 ARMADILLO; RRID: AB_528089
α-mCherry	Abcam	Abcam Cat# ab125096; RRID: AB_11133266
α-chicken AF488	Invitrogen	Molecular Probes Cat# A-11039; RRID: AB_142924
α-mouse AF568	Invitrogen	Molecular Probes Cat# A-21124; RRID: AB_141611

**Chemicals, Peptides, and Recombinant Proteins**		

Multisite Gateway Three Fragment vector construction kit	Invitrogen	12537023
Catalase	Sigma	C1345
Trypsin	Sigma	T1426
16% Methanol Free Paraformaldehyde	Alfa Aesar	11490570
Heptane	Sigma	34873
Triton X-100	Sigma	T8787
BSA	Sigma	A4503
Vectashield Mounting Media	Vector Labs	H-1000
Voltalef oil	VWR	24627.188
NLS-Cas9	NE Biolabs	M0646
RNase free water	Sigma	W4502
DNeasy Blood and Tissue Kit	QIAGEN	69504
MyTaq Red Mix	Meridian Bioscience	BIO-25043
BslI	NEBiolabs	R0555
MWoI	NEBiolabs	R0573
Tricaine/MS-22	Sigma	E10521
Horse serum	Sigma	H0146

**Critical commercial assays**		

Multisite Gateway Three Fragment vector construction kit	Invitrogen	12537023

**Experimental Models: Organisms/Strains**		

*DROSOPHILA*		N/A
*P{GawB}Pez*^*NP4748*^	Kyoto Stock Center	RRID: DGGR_104771
*serpentHemoGal4*	^23^	N/A
*serpentHemoGal4.2*	This work	N/A
*croquemort-Gal4*	^24^	N/A
*UAS-GFP*	BDSC	RRID: BDSC_6874
*UAS-2xeGFP*	BDSC	RRID: BDSC_6658
*UAS-PezTRiP*^*861*^	BDSC	RRID: BDSC_33918
*UAS-PezTRiP*^*862*^	BDSC	RRID: BDSC_33919
*UAS-TRiP*^*Luciferase*^	BDSC	RRID: BDSC_31603
*UAS-Pez*	^15^	N/A
*UAS-Pez*^*ΔFERM*^	^15^	N/A
*UAS-Pez*^*ΔPTP*^	^15^	N/A
*UAS-Pez*^*ΔPD*^	^15^	N/A
*UAS-Pez-sfGFP*	This work	N/A
*UAS-Pez-mCherry*	This work	N/A
*UAS-Draper-eGFP*	This work	N/A
*w*^*1118*^	BDSC	RRID: BDSC_3605
*Pez*^*CB*^	Kyoto Stock Center	RRID: DGGR_123596
*Pez^2^*	^15^	N/A
*src42A*^*[E1]*^	[25] BDSC	RRID: BDSC_6408
*draper*^*Δ5*^	^26^	N/A
*ZEBRAFISH*		N/A
*Tg(lysC:DsRed2)*	^23^	N/A
*Tg(mpeg:NLS-Scarlet)*^*ed207*^	This work	N/A
Tg(*lysC:NLS-mScarlet*)^ed229^	This work	N/A
Tg(*mpeg1,1:EGFP*)^gl22^	^27^	N/A

**Oligonucleotides**		

crRNA MEGF10 1: GCTACAGAACGGCCTATCGC	Sigma	Custom
crRNA MEGF10 2: TGTCAGTGTGAGCCGGGCTG	Sigma	Custom
crRNA PTPN21 1: GGTGGCATCATGTAGGGCTG	Sigma	Custom
crRNA PTPN21 2: GAATCAGGGCGCTGTGCCGG	Sigma	Custom
crRNA MEGF10 locus 1 genotyping fwd: aaccgaaaacaaatcaaaggagggc	Eurofins	Custom
crRNA MEGF10 locus 1 genotyping rev: acattgtaaaagcgctacagaaacaaa	Eurofins	Custom
crRNA MEGF10 locus 2 genotyping fwd: tgcttgtgtttgtttgcttg	Eurofins	Custom
crRNA MEGF10 locus 2 genotyping rev: tgaatggcttttgtcactcg	Eurofins	Custom
crRNA PTPN21 locus 1 genotyping fwd: gcagttcactataaaggcagc	Eurofins	Custom
crRNA PTPN21 locus 1 genotyping rev: gtggccgttaaagtgcatc	Eurofins	Custom
crRNA PTPN21 locus 2 genotyping fwd: gatgtcctccaacccaagca	Eurofins	Custom
crRNA PTPN21 locus 2 genotyping rev: aaaggatactgtcctgcgcc	Eurofins	Custom
tracrRNA	Sigma	TRACRRNA05N

**Software and Algorithms**		

GraphPad Prism V8.4.1	GraphPad Software	https://www.graphpad.com/scientific-software/prism/
ImageJ/FIJI	National Institute of Health	https://imagej.nih.gov/ij/
Volocity	PerkinElmer	https://www.perkinelmer.com/lab-products-and-services/resources/cellular-imaging-software-downloads.html
Zen Black	Zeiss	https://www.zeiss.com/microscopy/int/products/microscope-software/zen.html
Photoshop	Adobe	https://www.adobe.com/uk/products/photoshop.html
Illustrator	Adobe	https://www.adobe.com/uk/products/illustrator.html

### Resource Availability

#### Lead contact

Further information and requests for resources and reagents should be directed to and will be fulfilled by the Lead Contact, Will Wood w.wood@ed.ac.uk

#### Materials availability

Plasmids and transgenic lines generated in this study are available by request.

#### Data and code availability

This study did not generate datasets/code.

### Experimental Model and Subject Details

#### *Drosophila* stocks and genetics

*Drosophila* stocks were maintained according to standard protocols^26^. Embryos for live imaging and fixation were collected from apple juice agar plates from overnight laying cages (all incubated at 22°C, with the exception of RNAi experiments, which were kept at 29°C overnight to boost expression).

The following driver lines were combined with UAS constructs: *Pez-Gal4* (*P{GawB}Pez*^*NP4748*^*,* Kyoto), *serpentHemoGal4*^28^, *serpentHemoGal4.2* (*srp-Gal4.2*, an enhanced expression construct generated in the lab by Dr Kate Comber and Dr Fred Rodrigues) and *croquemort-Gal4* (*crq-Gal4;*^29^). The following UAS constructs were used in this study: *UAS-GFP, UAS-PezTRiP*^*861*^ (VDRC), *UAS-PezTRiP*^*862*^ *(*VDRC*), UAS-Pez, UAS-Pez*^*ΔFERM*^*, UAS-Pez*^*ΔPTP*^*, UAS-Pez*^*ΔPD*^ (all a kind gift of Dr. Hugo Stocker^17^), *UAS-Pez-sfGFP* and *UAS-Draper-eGFP* (both generated in this study – synthesized and cloned into pUASt by GeneArt and commercially injected by Best Gene Inc.). The mutant alleles used in this study were: *w*^*1118*^ (as a control background), *Pez*^*CB*^ (P{RS3} insert of 6.046 Kb - Kyoto), *Pez*^2^ (3083 bp deletion – line a gift from Dr. Hugo Stocker^17^), *src42A*^*[E1]*^ (EMS point mutant^30^) and *draper*^*Δ5*^ (1379 bp deletion^31^).

#### Zebrafish lines and rearing

All zebrafish lines were kept and raised under standard conditions^32^ and all experiments were approved by the British Home Office (project license No PEE579666). *Tg{lysC:DsRed2}*^23^ and *Tg(lysC:NLS-mScarlet)*^*ed229*^ lines were used to label neutrophils, whereas macrophages were visualized through *Tg*(*mpeg1,1:EGFP*)^gl2232^ and *Tg(mpeg1.1:NLS-mScarlet)*^ed207^. The *Tg(mpeg:NLS-Scarlet)*^*ed207*^ and *Tg*(*lysC:NLS-mScarlet*)^ed229^ line was generated using the Multisite Gateway Three Fragment vector construction kit (Invitrogen 12537023). In brief a 5′ Entry vector containing 1.85K mpeg1.1 promoter fragment or 8K lysC promoter (gift from Prof. Steve Renshaw), pME-nlsScarlet, p3E-(SV40)PolyA and a pDest-Tol2-polyA vector (Tol2 kit^33^) were added into a LR reaction according to manufacturer’s instruction. The recombinational cloning resulted in the final pDest-Tol2-mpeg1.1::nls-Scarlet-polyA and pDest-Tol2-lysC::nls-Scarlet-polyA vector. The final transgenic DNA plasmids were used to generate F0 founder fish. F1 adult fish was out crossed with wild-type fish, brightly labeled larvae were selected as F2. All experiments described were using F3 larvae from the F2 in cross.

### Method Details

#### Proteomics screen

Following overnight laying, stage 15 *w^∗^;srp-GAL4,UAS-GFP* and *w^∗^;src42A[E1],srp-Gal4,UAS-GFP* dechorionated embryos were collected in both the presence and absence of catalase. For the catalase treatment a 100x solution of 0.1 g catalase (Sigma C1345) in 1.9 mL of PBS was added to all solutions cells came into contact with. 250-280 embryos per sample were placed into the tip of a cold loose-fitting Dounce homogenizer. Embryos were then pestled gently in 250 μL of Seecof buffer.^34^ The pestle was washed with 750 μL dissociation media^34^ and transferred to an Eppendorf tube. The embryonic suspension was then sieved through at 40 μm nylon mesh and collected into a cold Eppendorf tube. The mixture was then centrifuged for 5 min at 350 r*cf.* at 4°C, the supernatant removed, and cells resuspended in 250 μL cold Seecof. Samples were kept on ice at all times.

Macrophages were then sorted by gating single/live/GFP+ cells into lysis buffer and kept at −80°C until further analysis. A total of 6 samples per treatment containing between 376,000-466,000 total cells were then pooled. Pooled samples were then trypsin (Sigma T1426) digested, and TMT labeled at the peptide level. All samples where then combined and phospho-enriched using a TiO2 column. Finally, phospho-enriched and TiO2 flow through (containing the non-phosphorylated peptides) were sent to LC-MS analysis. Returned peptide spectra were then compared to *Drosophila melanogaster* databases to obtain protein information. Ratios of peptide abundances were compared across sample type. Due to low overall protein abundance, the dataset was adjusted by normalizing to the median protein ratios of total protein levels between samples. See Figure S1.

#### *Drosophila* Fixation and immunostaining

Dechorionated embryos were collected in a 2 mL glass vial containing a 1:1 4% PFA:heptane mixture. Embryos were left tumbling in fixative for 30 minutes at room temperature, washed with PBS-Tx-BSA and incubated in primary antibodies at 4°C overnight. After washing with PBS-Tx-BSA and blocking with horse serum (2% v/v, Sigma-Aldrich) for 30 minutes, embryos were incubated with secondary antibodies for 1 hour at room temperature. Washed embryos were then mounted in Vectashield mounting medium. Primary antibodies: α-GFP (1:500, Abcam Ab13970), α-singed (Fascin, 1:100 DSHB Sn 7C) and α-armadillo (β catenin, 1:25, DSHB N2 7A1). Secondary antibodies: α-chicken AF488 (1:250 Invitrogen A11039) and α-mouse AF568 (1:250 Invitrogen A21124).

#### *Drosophila* Live imaging

Dechorionated embryos were staged and genotyped (by selecting against fluorescent balancer chromosomes) before being mounted in a droplet of VOLTALEF oil (VWR) on a glass slide, flanked by supporting coverslips with a bridging coverslip sealed on top as previously described.^35^ Images at z-slice intervals of 0.5 μm were acquired with a spinning disc confocal microscope (Perkin Elmer Ultraview) with either a 63x (NA 1.4) or a 40x (NA 1.3) objective. Epithelial wounds were generated by laser ablation as previously described^36^ using a nitrogen-pumped Micropoint ablation laser (Andor Technologies).

#### RNaseq data mining

Existing RNaseq datasets^21^^,^^22^ were accessed through Gene Expression Omnibus to mine for expression of PTPN21 and MEGF10 in 3 dpf larval neutrophils and 2 dpf larval macrophages. For both datasets, the raw counts matrix was used to calculate Transcripts Per Million (TPM) to account for sequencing depth and gene transcript length.

#### CRISPR-Cas9 gene editing of zebrafish embryos

CRISPR/Cas9-mediated mutant larvae “Crispants” were generated as described previously^24^. Briefly, CRISPR guide RNA (crRNA) sequences in which restriction enzyme recognition sequences overlapped the Cas9 cut site were identified in PTPN21 and MEGF10 exons and commercially synthesized (Sigma-Aldrich). 1 μL of each crRNAs were injected together into the embryo at the single-cell stage alongside 1 μL tracrRNA (Sigma-Aldrich), 0.3 μL NLS-Cas9 (NE Biolabs) and 1.7 μL RNase free water (Sigma-Aldrich). For neutrophil controls, Cas9 was omitted and replaced with a further 0.3 μL RNase free water. For macrophage experiments wound recruitment was compared to uninjected clutch-mates. Genotyping to confirm successful gene editing was performed following DNA extraction from individual larvae (95°C in 50mM NaOH for 1 hr, followed by addition of 0.5 M Tris-HCl pH 8.0) as previously described^24^). PCR of the edited region was performed using MyTaq Red Mix (Meridian Bioscience) and fragments were subsequently digested over night by the addition of 1 μL BslI or MwoI (NEBiolabs) directly to the reaction. Fragments were then resolved on a 2% agarose gel.

To quantify leukocyte numbers throughout the entire zebrafish larvae, control and Crispant fish were raised to 3 dpf and imaged using the VAST BioImager microscope platform as previously described^37^. Briefly, anesthetised live fish were mounted in glass capillaries and imaged laterally using a 1.6x post-magnification adaptor combined with a C-Plan-Apochromat 10x (NA 0.5) dipping lens (Carl Zeiss) and dual AxioCam 506 m CCD cameras (Carl Zeiss). Stitched maximum intensity projections of the entire larvae were imported into FIJI (NIH) and cell counter used to manually count fluorescent leukocyte nuclei.

#### Tailfin transection, fixation and staining

3 dpf larvae were anaesthetised by the addition of 0.02% buffered 3-aminobenzoic acid ethyl ester (Tricaine/MS-222) into the embryo medium and were left until paralysed. Using a scalpel, the entire tail fin and a small portion of the trunk distal to the end of the vasculature was removed. The embryos were then placed in fresh medium and allowed to recover. At 2 hours post injury (hpi), 6 hpi and 22 hpi larvae were culled using excess Tricaine. Culled larvae were then placed in an Eppendorf containing 4% PFA, 0.4% Triton-X diluted in PBS and fixed overnight at 4°C or at room temperature for 2 hours.

Wholemount immunostaining of zebrafish larvae was performed as described previously^38^. Wash buffer comprising PBS containing 0.1% Triton-X (PBST Sigma-Aldrich) and 5% horse serum (Sigma Aldrich) was used for blocking. Both primary and secondary antibodies were diluted in PBST containing 2%–5% horse serum and were left to incubate over night at 4°C. DAPI was added to secondary antibodies to visualize the entire tissue. Primary antibody: α-mCherry (1:500, Abcam Ab125096). Secondary antibody: α-mouse AF 568 (1:250 Invitrogen A21124). Stained samples were mounted laterally in Vectashield on glass slides and imaged on a Zeiss LSM880 confocal microscope using a 25x objective (NA 0.8).

### Quantification and Statistical Analysis

All images were imported into FIJI (NIH). Vacuoles were counted in raw images before z-projection as fluorescent negative areas within the cell body. For cell tracking, the manual tracking plugin was used, and data was exported to Microsoft Excel to obtain mean cell speed and distance traveled. Meandering index was calculated as Euclidean distance/Total distance traveled and responding cells were defined as those that reached the wound site at any point within 2 hr. To quantify macrophages recruited to wounds in *Drosophila* embryos, the outline of the wound was defined using bright field images and was then drawn across all Z slices. Inflammatory recruitment was defined as any macrophage that contacted (specifically via its cell body) the wound perimeter over the time course of imaging following wounding. For wound recruitment analysis, macrophage numbers recruited to wounds in *Drosophila* embryos were divided by the wound perimeter to account for differing wound sizes due to variation in laser ablation. To quantify wound closure, wound perimeter was recorded over time and analyzed as a function of wound size at 10 minutes – the earliest time point at which the wound outline can be accurately measured by brightfield imaging.

Following live imaging of fluorescently tagged constructs within macrophages, lamellipods were outlined manually for visualization using the Freehand selection tool. Puncta were tracked using manual tracking and counted using the cell counter plugin within FIJI (NIH). Kymographs were generated using the reslice tool along a line (10 pixels wide) following the path of an individual punctum (line drawn using segmented line tool to accommodate non-linear path of the puncta). In each kymograph, the x axis represents distance starting at the lamellipod leading edge on the left, toward the cell body on the right (174 nm/pixel, 17.4 μm total). The y axis represent time (10 s/pixel, 2.5 min total).

For quantification of zebrafish larvae tailfin transection, a 150 μm area was outlined extending from the wound margin. All DsRed2/mScarlet positive leukocytes within this area were counted manually.

Raw data was collated using Microsoft Excel and imported into Prism 8 (GraphPad) for statistical analysis and graphing. All datasets underwent Normality tests to ensure the appropriate statistical tests were performed. For normally distributed data Unpaired t tests were performed, with Welch’s correction where variances were significantly different (determined by F-test). For data not normally distributed, Mann-Whitney U tests were performed to confer significance. ANOVA tests were performed for datasets with more than two groups for comparison. For data with comparable variances (F-tested) Tukey’s or Sidak’s multiple comparisons were performed as recommended by the software. Brown-Forsythe and Welch ANOVA tests were used were variances significantly differed.

## Supplemental Information

Document S1. Figures S1 and S2

Document S2. Article plus Supplemental Information

## References

[1] Moreira S., Stramer B., Evans I., Wood W., Martin P. (2010). Prioritization of competing damage and developmental signals by migrating macrophages in the Drosophila embryo. Curr. Biol..

[2] Razzell W., Evans I.R., Martin P., Wood W. (2013). Calcium flashes orchestrate the wound inflammatory response through DUOX activation and hydrogen peroxide release. Curr. Biol..

[3] Evans I.R., Rodrigues F.S.L.M., Armitage E.L., Wood W. (2015). Draper/CED-1 mediates an ancient damage response to control inflammatory blood cell migration in vivo. Curr. Biol..

[4] Weavers H., Liepe J., Sim A., Wood W., Martin P., Stumpf M.P.H. (2016). Systems analysis of the dynamic inflammatory response to tissue damage reveals spatiotemporal properties of the wound attractant gradient. Curr. Biol..

[5] Wood W., Martin P. (2017). Macrophage functions in tissue patterning and disease: new insights from the fly. Dev. Cell.

[6] Yoo S.K., Starnes T.W., Deng Q., Huttenlocher A. (2011). Lyn is a redox sensor that mediates leukocyte wound attraction in vivo. Nature.

[7] Niethammer P., Grabher C., Look A.T., Mitchison T.J. (2009). A tissue-scale gradient of hydrogen peroxide mediates rapid wound detection in zebrafish. Nature.

[8] de Oliveira S., López-Muñoz A., Candel S., Pelegrín P., Calado Â., Mulero V. (2014). ATP modulates acute inflammation in vivo through dual oxidase 1-derived H2O2 production and NF-κB activation. J. Immunol..

[9] Katikaneni A., Jelcic M., Gerlach G.F., Ma Y., Overholtzer M., Niethammer P. (2020). Lipid peroxidation regulates long-range wound detection through 5-lipoxygenase in zebrafish. Nat. Cell Biol..

[10] Møller N.P., Møller K.B., Lammers R., Kharitonenkov A., Sures I., Ullrich A. (1994). Src kinase associates with a member of a distinct subfamily of protein-tyrosine phosphatases containing an ezrin-like domain. Proc. Natl. Acad. Sci. USA.

[11] Cardone L., Carlucci A., Affaitati A., Livigni A., DeCristofaro T., Garbi C., Varrone S., Ullrich A., Gottesman M.E., Avvedimento E.V., Feliciello A. (2004). Mitochondrial AKAP121 binds and targets protein tyrosine phosphatase D1, a novel positive regulator of src signaling. Mol. Cell. Biol..

[12] Carlucci A., Gedressi C., Lignitto L., Nezi L., Villa-Moruzzi E., Avvedimento E.V., Gottesman M., Garbi C., Feliciello A. (2008). Protein-tyrosine phosphatase PTPD1 regulates focal adhesion kinase autophosphorylation and cell migration. J. Biol. Chem..

[13] Zanet J., Stramer B., Millard T., Martin P., Payre F., Plaza S. (2009). Fascin is required for blood cell migration during Drosophila embryogenesis. Development.

[14] Tepass U., Fessler L.I., Aziz A., Hartenstein V. (1994). Embryonic origin of hemocytes and their relationship to cell death in Drosophila. Development.

[15] Cho N.K., Keyes L., Johnson E., Heller J., Ryner L., Karim F., Krasnow M.A. (2002). Developmental control of blood cell migration by the Drosophila VEGF pathway. Cell.

[16] Wood W., Faria C., Jacinto A. (2006). Distinct mechanisms regulate hemocyte chemotaxis during development and wound healing in Drosophila melanogaster. J. Cell Biol..

[17] Poernbacher I., Baumgartner R., Marada S.K., Edwards K., Stocker H. (2012). Drosophila Pez acts in Hippo signaling to restrict intestinal stem cell proliferation. Curr. Biol..

[18] Weavers H., Evans I.R., Martin P., Wood W. (2016). Corpse engulfment generates a molecular memory that primes the macrophage inflammatory response. Cell.

[19] Chishti A.H., Kim A.C., Marfatia S.M., Lutchman M., Hanspal M., Jindal H., Liu S.-C., Low P.S., Rouleau G.A., Mohandas N. (1998). The FERM domain: a unique module involved in the linkage of cytoplasmic proteins to the membrane. Trends Biochem. Sci..

[20] Williamson A.P., Vale R.D. (2018). Spatial control of Draper receptor signaling initiates apoptotic cell engulfment. J. Cell Biol..

[21] Kenyon A., Gavriouchkina D., Zorman J., Napolitani G., Cerundolo V., Sauka-Spengler T. (2017). Active nuclear transcriptome analysis reveals inflammasome-dependent mechanism for early neutrophil response to Mycobacterium marinum. Sci. Rep..

[22] Kuil L.E., Oosterhof N., Ferrero G., Mikulášová T., Hason M., Dekker J., Rovira M., van der Linde H.C., van Strien P.M., de Pater E. (2020). Zebrafish macrophage developmental arrest underlies depletion of microglia and reveals Csf1r-independent metaphocytes. eLife.

[23] Hall C., Flores M.V., Storm T., Crosier K., Crosier P. (2007). The zebrafish lysozyme C promoter drives myeloid-specific expression in transgenic fish. BMC Dev. Biol..

[24] Keatinge M., Tsarouchas T.M., Munir T., Larraz J., Gianni D., Tsai H.-H., Becker C.G., Lyons D.A., Becker T. (2020). Phenotypic screening using synthetic CRISPR gRNAs reveals pro-regenerative genes in spinal cord injury. bioRxiv.

[25] Renshaw S.A., Loynes C.A., Trushell D.M.I., Elworthy S., Ingham P.W., Whyte M.K.B. (2006). A transgenic zebrafish model of neutrophilic inflammation. Blood.

[26] Greenspan R.J. (1997). Fly Pushing: The Theory and Practice of Drosophila Genetics 2^nd^ Revision.

[27] Ellett F., Pase L., Hayman J.W., Andrianopoulos A., Lieschke G.J. (2011). mpeg1 promoter transgenes direct macrophage-lineage expression in zebrafish. Blood.

[28] Brückner K., Kockel L., Duchek P., Luque C.M., Rørth P., Perrimon N. (2004). The PDGF/VEGF receptor controls blood cell survival in Drosophila. Dev. Cell.

[29] Stramer B., Wood W., Galko M.J., Redd M.J., Jacinto A., Parkhurst S.M., Martin P. (2005). Live imaging of wound inflammation in Drosophila embryos reveals key roles for small GTPases during in vivo cell migration. J. Cell Biol..

[30] Tateno M., Nishida Y., Adachi-Yamada T. (2000). Regulation of JNK by Src during Drosophila development. Science.

[31] Freeman M.R., Delrow J., Kim J., Johnson E., Doe C.Q. (2003). Unwrapping glial biology: Gcm target genes regulating glial development, diversification, and function. Neuron.

[32] Westerfield M. (2007). The Zebrafish Book. A Guide for the Laboratory Use of Zebrafish (Danio rerio).

[33] Kwan K.M., Fujimoto E., Grabher C., Mangum B.D., Hardy M.E., Campbell D.S., Parant J.M., Yost H.J., Kanki J.P., Chien C.-B. (2007). The Tol2kit: a multisite gateway-based construction kit for Tol2 transposon transgenesis constructs. Dev. Dyn..

[34] Seecof R.L., Alléaume N., Teplitz R.L., Gerson I. (1971). Differentiation of neurons and myocytes in cell cultures made from Drosophila gastrulae. Exp. Cell Res..

[35] Evans I.R., Zanet J., Wood W., Stramer B.M. (2010). Live imaging of Drosophila melanogaster embryonic hemocyte migrations. J. Vis. Exp..

[36] Wood W., Jacinto A., Grose R., Woolner S., Gale J., Wilson C., Martin P. (2002). Wound healing recapitulates morphogenesis in Drosophila embryos. Nat. Cell Biol..

[37] Early J.J., Cole K.L.H., Williamson J.M., Swire M., Kamadurai H., Muskavitch M., Lyons D.A. (2018). An automated high-resolution in vivo screen in zebrafish to identify chemical regulators of myelination. eLife.

[38] Feng Y., Santoriello C., Mione M., Hurlstone A., Martin P. (2010). Live imaging of innate immune cell sensing of transformed cells in zebrafish larvae: parallels between tumor initiation and wound inflammation. PLoS Biol..

