# Supplementary material for: PTPN21/Pez Is a Novel and Evolutionarily Conserved Key Regulator of Inflammation In Vivo
Source: Curr Biol. 2021 Feb 22;31(4):875–883.e5. doi: 10.1016/j.cub.2020.11.014 (PMC7902905; doi:10.1016/j.cub.2020.11.014)
Supplement: Document S1. Figures S1 and S2 [file mmc1.pdf]

Current Biology, Volume *31*

Supplemental Information

PTPN21/Pez Is a Novel and Evolutionarily Conserved Key Regulator of Inflammation *In Vivo*

Jennie S. Campbell, Andrew J. Davidson, Henry Todd, Frederico S.L.M. Rodrigues, Abigail M. Elliot, Jason J. Early, David A. Lyons, Yi Feng, and Will Wood

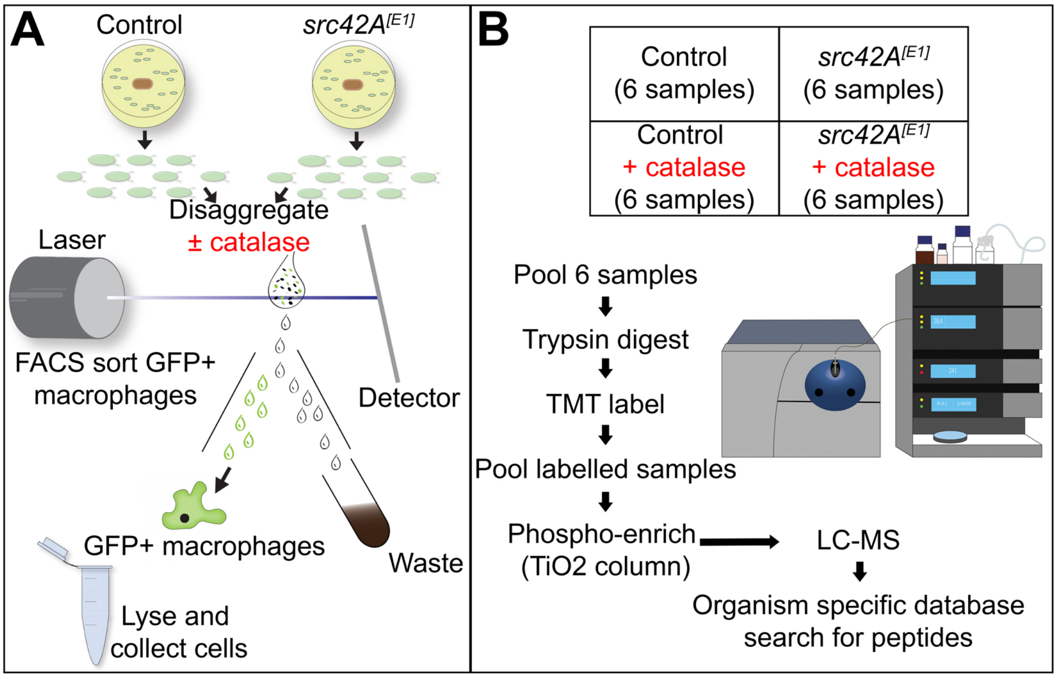

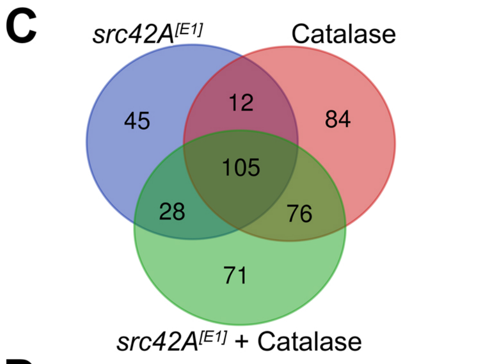

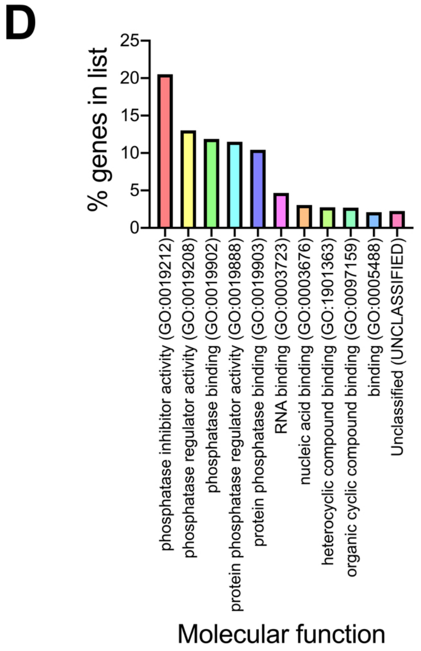

E

Accession	Name	Brief function	*src42A[E1]* raw	*src42A[E1]* normalised	Catalase raw	Catalase normalised	*src42A[E1]* +catalase raw	*src42A[E1]* +catalase normalised
O16125	Bifocal	Actin and microtubule binding	0.189	0.119	0.902	0.506	0.189	0.136
Q9VUY9	PGM	Glucose metabolism	0.479	0.301	0.479	0.268	0.479	0.344
Q0KHY3	Mesh	Smooth septate junction organisation	0.507	0.319	1.306	0.732	0.583	0.419
Q9NCL3	Mud	Spindle orientation	0.540	0.340	1.058	0.593	0.789	0.567
Q9VYW4	CG1703	ATP binding	0.588	0.370	1.306	0.732	0.641	0.460
Q9U1J9	SF1	mRNA splicing	0.666	0.419	1.096	0.614	0.796	0.572
O01349	Khc-73	Kinesin heavy chain	0.669	0.421	2.402	1.346	0.736	0.529
A0A0B4LHK4	Mask	Protein kinase binding activity/RNA binding	0.684	0.430	2.022	1.133	1.460	1.049
Q6NP56	SRPK	Spindle microtubule assembly	0.685	0.431	1.015	0.569	1.843	1.324
Q7YU13	Polybromo	Chromatin remodeling	0.725	0.456	1.051	0.589	0.510	0.367
Q9V3V3	Pez	Regulation of Hippo signalling	0.730	0.459	0.495	0.277	1.510	1.085
Q7K4B6	Tmtc3	Mannosylation	0.761	0.479	1.161	0.651	0.478	0.343
Q2XXV4	mus101	DNA replication checkpoint	0.766	0.482	1.017	0.570	0.679	0.488
Q9VBN5	RpL27	Structural constituent of ribosome	0.772	0.486	1.364	0.765	0.873	0.627
Q95TP9	RpS6	Structural constituent of ribosome	0.817	0.514	1.631	0.914	0.729	0.524
Q9VLT1	MON2	Cytoskeletal regulation	0.838	0.527	1.289	0.723	0.492	0.354
Q06943	HmgZ	Chromatin remodelling/DNA binding	0.840	0.529	1.980	1.110	1.142	0.820
Q7K4H1	CG8185	Secreted protein	0.858	0.540	0.804	0.451	1.302	0.936
Q9U5L1	Gtp-bp	Component of the signal recognition particle receptor	0.877	0.552	0.877	0.492	0.877	0.630
Q95RH0	Msk	Protein import into the nucleus	0.880	0.554	4.581	2.568	2.845	2.044

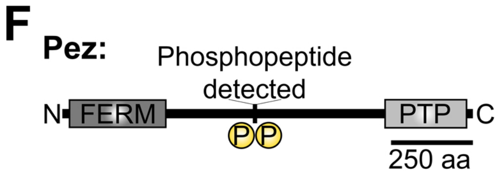

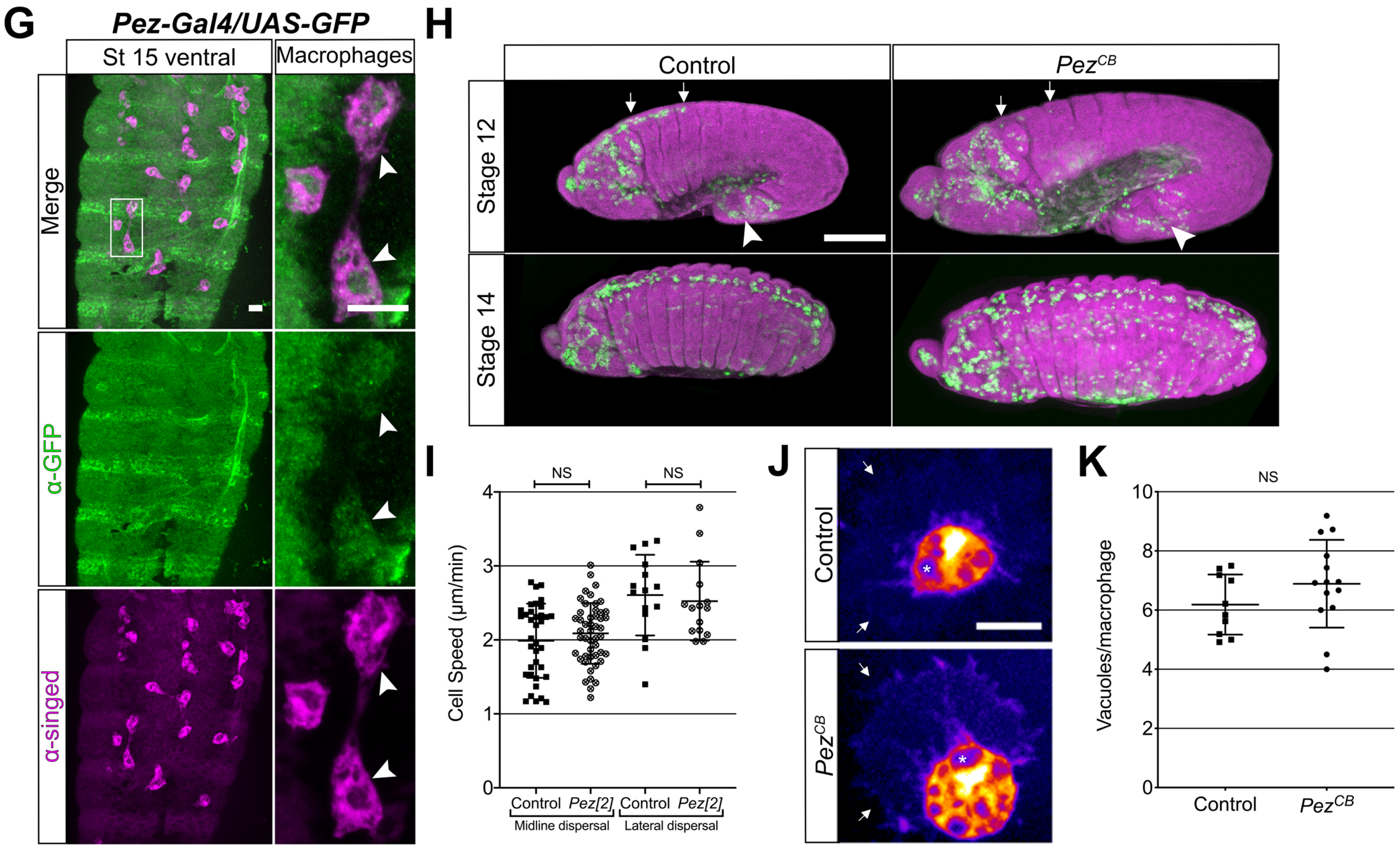

Figure S1: A phosphoproteomics screen reveals Pez is indirectly phosphorylated following H_2O_2 signalling and Src42A activation. Pez is dispensable for basal macrophage behaviours. Related to STAR Methods and Video S1.

A) Schematic to show collection of GFP+ macrophages from both control and *src42A* mutant embryos. Embryos from both genotypes were collected and disaggregated in the presence and absence of catalase to quench H_2O_2 signalling. The crushed embryos were sorted by FACS and GFP positive macrophages were collected, lysed and frozen. B) In total 6 samples for each condition were collected. These were pooled, digested with trypsin and differentially TMT labelled per condition. All samples were then pooled, before being sent for phosphoenrichment. Phosphopeptides were then detected by liquid chromatography mass spectrometry (LC-MS), and results were mapped back to proteins following an organism-specific database search. C) Venn diagram to show the number of individual phosphopeptides with levels reduced by >30% in each condition (*src42A* mutant in blue, catalase treatment in red and *src42A* mutant + catalase treatment in green). Treatment circles overlapping where reduction was detected in multiple conditions. In total 421 phosphopeptides were depleted by more than 30% following the abolition of the H_2O_2-Src42A signalling axis. D) PANTHER Go analysis of depleted phosphopeptides reveals an enrichment in phosphatase related activities in list in comparison to representation across *Drosophila* genome. E) Top 20 hits from proteomics analysis, displayed with accession number and name. Raw values for protein abundance in comparison to control, as well as value normalised to median protein levels for the sample. Green denotes fold change below 20%. Pez – a PTP type phosphatase is outlined in orange. F) Pez, is phosphorylated in the central linker region (denoted by P in yellow circles) on two serine residues downstream of H_2O_2 and Src42A. Schematic depicts N terminal

FERM domain and C terminal PTP domain of Pez. G) Ventral view of *Pez-Gal4* x *UAS-GFP* stage 15 embryo. α-GFP staining (green) reveals expression of Pez throughout the embryo. Co-staining with α-singed (Fascin, magenta) demonstrates *Pez* expression within macrophages (arrowheads). Scale bars = 10 μm. H) Macrophages visualised during developmental migration (*Serpent-Gal4.2,UAS-2xeGFP,* α-GFP staining (Green)) at stage 12 and 14 reveal Pez^{CB} mutant macrophages follow stereotypical routes, migrating into the germ band (arrowheads) and along the ventral nerve cord (arrows). Scale bars = 100 μm. I) Cell migratory speeds of control and Pez^{2} mutant macrophages undergoing both midline and lateral dispersal are comparable (n ≥36 (midline) and n =15 (lateral) cells from ≥3 embryos/genotype, Mann-Whitney U test). J) Macrophages expressing eGFP (Fire LUT used to overcome saturating fluorescence in cell body). Like control cells, Pez^{CB} mutant macrophage display lamellipodial protrusions (arrows) and intracellular vacuoles (asterisks). K) Pez^{CB} mutant macrophages contain comparable numbers of intracellular vacuoles to controls (n ≥65 cells from ≥3 embryos/genotype, Mann-Whitney U test).

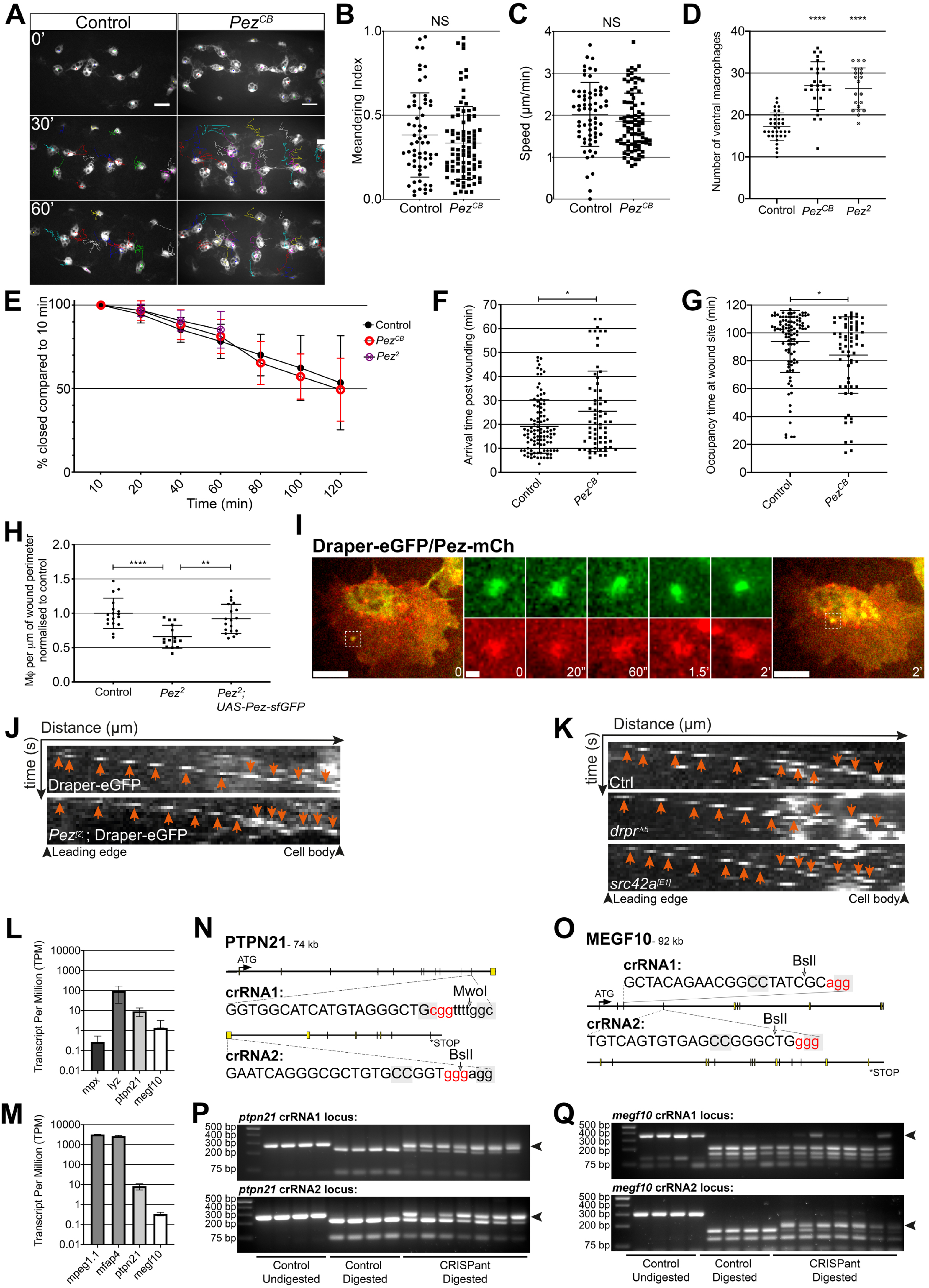

A
Control
PezCB
0'
30'
60'
B
NS
Meandering Index
Control
PezCB
C
NS
Speed (µm/min)
D
****
Number of ventral macrophages
Pez2
E
% closed compared to 10 min
Time (min)
Control
PezCB
Pez2
F
Arrival time post wounding (min)
G
Occupancy time at wound site (min)
H
Mϕ per µm of wound perimeter normalised to control
Pez2; UAS-Pez-sfGFP
I
Draper-eGFP/Pez-mCh
J
Distance (µm)
time (s)
Draper-eGFP
Pez[2]; Draper-eGFP
Leading edge
Cell body
K
Ctrl
drprΔ5
src42a[E1]
L
Transcript Per Million (TPM)
mpx
lyz
ptpn21
megf10
M
mpeg1.1
mfap4
N
PTPN21- 74 kb
ATG
crRNA1:
MwoI
GGTGGCATCATGTAGGGCTGcggttttggc
*STOP
crRNA2:
BslI
GAATCAGGGCGCTGTGCCGGTgggagg
O
MEGF10- 92 kb
crRNA1:
GCTACAGAACGGCCTATCGCagg
crRNA2:
TGTCAGTGTGAGCCGGGCTGggg
P
ptpn21 crRNA1 locus:
ptpn21 crRNA2 locus:
Control Undigested
Control Digested
CRISPant Digested
Q
megf10 crRNA1 locus:
megf10 crRNA2 locus:

Figure S2: Pez drives the inflammatory recruitment of macrophages to wounds. Related to Video S2, Video S3 and Figures 1, 3 and 4.

A) Cell tracks over 1 hour for control and *Pez*^{CB} mutant ventral macrophages during their basal (unwounded) migration. Scale bars =20 μm. Analysis of macrophage tracks reveal no difference in basal B) meandering index or C) migratory speed (n ≥65 cells from 5 embryos/genotype, Mann-Whitney U test). D) Analysis of ventral macrophage numbers at stage 15 reveals a significant increase in cell numbers in both *Pez* mutants in comparison to control (n ≥20 embryos/genotype, one-way ANOVA with Dunnet's multiple comparisons). E) The closure rate of wounds calculated as percentage closure compared to the wound size at 10 minutes showing no significant differences in closure rate in either *Pez* mutant in comparison to control (n ≥10 wounded embryos/genotype, multiple t test). Mean and standard deviation plotted. F) *Pez* mutant macrophages reach the wound site at later timepoints in comparison to control and thus (G) spend significantly less time at the site of damage (n=53 responding cells from ≥5 embryos/genotype, Mann-Whitney U tests). H) UAS-Pez-sfGFP is sufficient to rescue the suppressed inflammatory macrophage recruitment of *Pez*^{2} mutant macrophages (≥5 wounded embryos/genotype, one way ANOVA with Holm-Sidak multiple comparisons). All error bars are mean ±SD. NS, not significant, *p<0.05, **p<0.01, ****p<0.001. I) Representative images to show colocalization of Draper-eGFP and Pez-mCherry at a wound-induced puncta. Cell imaged over 2 minutes post wounding. Boxed region expanded in intervening images (moved relative to punctum). Scale bars =10/1 μm. J) Kymographs showing wound-induced Draper-eGFP clusters (orange arrows) in control and *Pez*^{2} mutant macrophages K) Kymography demonstrates that wound-induced, individual Pez-sfGFP puncta (orange arrows) in control, draper or src42A mutant macrophages have very similar dynamics. For all

kymographs, the x-axes represent distance starting at lamellipod leading edge (174 nm/pixel, 17.4 μm total). The y-axes represent time (10 s/pixel, 2.5 min total). L) RNAseq data mining reveals ptpn21 and megf10 are expressed in 3 dpf zebrafish larval neutrophils alongside myeloperoxidase (mpx) and lysozyme (lyz). Data plotted as transcript per million on a logarithmic scale. M) Similarly, both ptpn21 and megf10 are expressed alongside mpeg1.1 and mfap4 in macrophage in 2 dpf zebrafish. N) Schematic highlighting crRNA target sites (capitals) within the PTPN21 locus (74 kb). PAM sites (red) were chosen to disrupt restriction enzyme digestion sites MwoI/BslI for validation by PCR. Exons represented by yellow rectangles. O) MEGF10 locus (92kb) with CRISPR target sites at BslI restriction enzyme sites highlighted. crRNA sequences in capitals and PAM site denoted in red. P) PCR amplification of the target loci within *ptpn21* gene. Fragments from control injected fish show complete digestion. Arrows highlight bandshifts arising from successful, CRISPR-based modification. Q) PCR amplification of the target loci within *megf10* gene. Fragments from control injected fish show complete digestion (resulting in multiple banding). Crispant fish (arrowheads) demonstrate incomplete digestion and therefore efficient editing of the locus. The cRNA2 locus fragment contains two BslI sites – one of which is edited following injection. Arrows highlight bandshifts arising from successful, CRISPR-based modification.
